# Effectivity of pazopanib treatment in orthotopic models of human testicular germ cell tumors

**DOI:** 10.1186/1471-2407-13-382

**Published:** 2013-08-10

**Authors:** Mercè Juliachs, August Vidal, Xavier Garcia del Muro, Josep M Piulats, Enric Condom, Oriol Casanovas, Mariona Graupera, Jose R Germà, Alberto Villanueva, Francesc Viñals

**Affiliations:** 1 Laboratori de Recerca Translacional, Institut Català d'Oncologia, Hospital Duran i Reynals, L’Hospitalet de Llobregat, Barcelona, 08908, Spain; 2 Servei d’Oncologia Mèdica, Institut Català d’Oncologia, Hospital Duran i Reynals, L’Hospitalet de Llobregat, Barcelona, 08908, Spain; 3 Servei d’Anatomia Patològica, Hospital Universitari de Bellvitge, L’Hospitalet de Llobregat, Barcelona, 08908, Spain; 4 Laboratori d’Oncologia Molecular, Institut d’Investigació Biomèdica de Bellvitge (IDIBELL), L’Hospitalet de Llobregat, Barcelona, 08908, Spain; 5 Departament de Ciències Fisiològiques II, Universitat de Barcelona, L’Hospitalet de Llobregat, Barcelona, 08908, Spain; 6 Departament de Patologia i Terapèutica Experimental, Universitat de Barcelona, L’Hospitalet de Llobregat, Barcelona, 08908, Spain; 7 Institut d’Investigació Biomèdica de Bellvitge (IDIBELL), L’Hospitalet de Llobregat, Barcelona, 08908, Spain

**Keywords:** Pazopanib, Lapatinib, Testicular cancer, Germ-cell tumors, Cisplatin, Refractory

## Abstract

**Background:**

Cisplatin (CDDP) resistance in testicular germ cell tumors (GCTs) is still a clinical challenge, and one associated with poor prognosis. The purpose of this work was to test pazopanib, an anti-tumoral and anti-angiogenic multikinase inhibitor, and its combination with lapatinib (an anti-ErbB inhibitor) in mouse orthotopic models of human testicular GCTs.

**Methods:**

We used two different models of human testicular GCTs orthotopically grown in nude mice; a CDDP-sensitive choriocarcinoma (TGT38) and a new orthotopic model generated from a metastatic GCT refractory to first-line CDDP chemotherapy (TGT44). Nude mice implanted with these orthotopic tumors were treated with the inhibitors and the effect on tumoral growth and angiogenesis was evaluated.

**Results:**

TGT44 refractory tumor had an immunohistochemical profile similar to the original metastasis, with characteristics of yolk sac tumor. TGT44 did not respond when treated with cisplatin. In contrast, pazopanib had an anti-angiogenic effect and anti-tumor efficacy in this model. Pazopanib in combination with lapatinib in TGT38, an orthotopic model of choriocarcinoma had an additive effect blocking tumor growth.

**Conclusions:**

We present pazopanib as a possible agent for the alternative treatment of CDDP-sensitive and CDDP-refractory GCT patients, alone or in combination with anti-ErbB therapies.

## Background

Germ cell tumors (GCTs) of the testis are an uncommon malignancy, but constitute the most frequent cancer type among men aged between 15 and 35 years [1]. GCTs can be divided into seminoma or non-seminoma tumors on the basis of histological, biological and clinical features. Non-seminoma GCTs may consist of several distinct histological components (such as teratoma, embryonal carcinoma, yolk sac tumor and choriocarcinoma) or combinations thereof [2], and while almost all seminomas are curable with orchiectomy, non-seminomas frequently require chemotherapy and surgery, and are less sensitive to radiotherapy [3]. Excellent cure rates have been achieved even in metastatic testicular cancer, and more than 70% of these patients achieve a complete response with first-line chemotherapy based on CDDP, alone or combined with surgery. However, some patients do have late relapses, which are usually chemotherapy-resistant, or refractory diseases following their first-line chemotherapy. Treatment of these patients consists in most cases of second-line CDDP-based chemotherapy and radical surgery, which only occasionally produces durable responses [3-6]. Therefore, new alternative therapies for refractory and resistant patients are needed.

Angiogenesis, the recruitment of new blood vessels, is essential for tumor growth and metastasis, and is driven by a balance between anti-angiogenic and pro-angiogenic factors. VEGF and PDGF are two of several molecules that promote angiogenesis by binding to specific cell-surface tyrosine kinase receptors (TKRs) [7,8]. Anti-angiogenic therapies have shown efficacy in the treatment of various tumor types, directly targeting VEGF (such as the antibody bevacizumab) as well as the combined inhibition of VEGFRs and PDGFRs by multitarget tyrosine kinase inhibitors (TKIs) [9,10]. Testicular GCTs usually have vascular invasion [11], and previous studies have described the involvement of c-KIT, PDGFRs, VEGFRs and their ligands in the tumorigenesis of the GCTs of the testis [11-15].

Pazopanib (GW786034) is an oral multikinase inhibitor that targets the TKRs VEGFR1, VEGFR2, VEGFR3, PDGFRα, PDGFRβ and c-KIT [16,17]. Pre-clinical *in vivo* studies of pazopanib have shown it to inhibit VEGF-induced angiogenesis, tumor angiogenesis and the growth of several human tumor xenografts (multiple myeloma, colon, melanoma, prostate, kidney, breast and lung tumors) in mice [16,18]. Pazopanib has been shown to have significant clinical benefit in several phase II and III studies in a wide variety of malignancies, including soft tissue sarcoma, thyroid cancer, ovarian cancer, non-small cell lung cancer [19-23], and in patients with metastatic renal cell carcinoma (RCC) [19,24]. Pazopanib was approved by the US FDA for the treatment of patients with advanced RCC in 2009 [25] and was conditionally approved by the European Medicines Agency in 2010.

In the present study we evaluate the efficacy of pazopanib in two models of human testicular GCTs orthotopically grown in nude mice: a CDDP-sensitive choriocarcinoma and a new orthotopic model originated from a metastatic GCT that is refractory to first-line CDDP chemotherapy. Moreover we tested pazopanib alone or in combination with the anti-ErbB inhibitor lapatinib.

## Methods

### Chemical compounds

Pazopanib (Votrient®) and Lapatinib (Tyverb®) were provided by GlaxoSmithKline. Both were dissolved in 0.5% carboxymethylcellulose – 0.1% Tween 80 (Sigma) solution. CDDP was provided by the Pharmacological Department of our institution; it was diluted in sterile serum before intraperitoneal injection. Drug aliquots were prepared once weekly and kept in the dark at 4°C.

### Orthotopic implantation of testicular tumors

Male nu/nu Swiss mice were purchased from Harlan (Spain). Mice were housed and maintained in laminar flow cabinets under specific pathogen-free conditions. All the animal studies were approved by the local committee for animal care (IDIBELL, Ref. PR218/09).

The testicular GCTs used were perpetuated in nude mice by consecutive passages. We used two orthotopic testicular GCTs models for our studies; a choriocarcinoma (TGT38), previously described by Castillo-Avila et al. [26], and TGT44, originated from a human retroperitoneal metastatic mixed GCT with teratoma and yolk sac components. This tumor was originally refractory to first-line CDDP chemotherapy, and the yolk sac component is able to grow in nude mice.

For the surgical implantation, mice were anesthetized by isoflurane inhalation. A small midline incision was made and the testes were exteriorized. A piece of 2–4 mm^3^ tumor was implanted in each testis using Prolene 7.0 surgical sutures. The testes were returned to the abdominal cavity and the incision was closed with wound clips. Meloxicam was administered subcutaneously to the mice (5 mg/kg) the day of the surgical intervention and for two days after implantation.

For the first two passages of TGT44, mice bearing this orthotopic tumor were treated with three doses of 4 mg/kg CDDP as a first CDDP resistance test. No difference in time of tumoral growth was observed between CDDP-treated mice and vehicle-treated mice.

### Treatment schedule

As the tumors had different growth behaviors the treatment schedules were different for TGT38 and TGT44. For both tumors, treatments started when a palpable intra-abdominal mass was detected; studies were terminated when tumors in vehicle-treated animals were judged to be adversely affecting their wellbeing.

The treatment of mice bearing the TGT44 tumor started six weeks after tumor implantation and continued for six more weeks. Four mice were treated with pazopanib, administered daily with gavage as an oral dose of 100 mg/kg [16], while oral vehicle solution was administered daily by gavage to the control group (three mice). Three mice were treated with four doses of 4 mg/kg CDDP, administered intraperitoneally once a week for the first four weeks. Control group mice received intraperitoneal sterile serum with the same schedule as CDDP mice.

Regarding TGT38 tumor, treatment started 13 days after tumor implantation. Twelve mice were treated with pazopanib, administered daily with an oral dose of 100 mg/kg, as previously described by Kumar et al. [16]. Thirteen mice were treated daily with 100 mg/kg lapatinib, administered orally [27]. For the pazopanib/lapatinib combination group, twelve animals were treated daily with pazopanib (100 mg/kg) and lapatinib (100 mg/kg), administered orally. Eighteen mice were treated with vehicle oral solution with the same schedule as the treated groups. Mice were treated for 14 days.

These treatments had no significant effect on mouse body weight and the animals appeared healthy and active throughout the study. Mice were sacrificed by CO_2_ inhalation and the effects of the different treatments on tumor response were evaluated by determining tumor weight and volume, where volume=(length)(width^2^/2). In order to show whether single and combined treatments have toxic effect, an apoptotic cell analysis in liver was perfomed in control and treated mice. The results obtained showed lack of toxic effects of all treatments.

To examine the possible synergy between lapatinib and pazopanib in the combination treatment group, we calculated the combination ratio (CR), as described elsewhere [28]. The fractional tumor volume (FTV) for each treatment group was calculated as the ratio of the mean volumes of treated to control tumors, giving values for FTV_lapatinib_, FTV_pazopanib_ and FTV_lapatinib+pazopanib_. The expected FTV for the combination group was defined as observed FTV_lapatinib_ x observed FTV_pazopanib._ The ratio of expected FTV_lapatinib+pazopanib_ / observed FTV_lapatinib+pazopanib_ is the CR. We concluded that values of CR>1 indicated supra-additive effects, while values of CR<1 indicated infra-additive effects.

### Immunofluorescence studies

OCT-frozen tissue sections (3 μm) from control and pazopanib-treated tumors were used for immunofluorescence vessel staining. Sections were fixed with 4% paraformaldehyde for 10 min and then washed once with distilled water and twice with PBS 0.1% Triton X-100. These were then incubated overnight at 4°C with a 1:50 dilution of rat monoclonal antibody for CD31 (BD Pharmingen). Sections were washed twice with PBS 0.1% Triton X-100 and incubated with a 1:200 dilution of Alexa Fluor 488-conjugated goat anti-rat at room temperature for 1 h in the dark. TGT38 tumor slides were washed twice in PBS 0.1% Triton X-100 and incubated with a 1:1000 dilution of TO-PRO-3 (Molecular Probes) for 30 min in the dark. Finally, the slides were washed twice in PBS, and coverslips were mounted using Gel Mount aqueous mounting medium (Sigma). TGT44 tumor sections were mounted using VectaShield mounting medium for fluorescence with DAPI.

Images of TGT38 sections were obtained on a Leica TCS SL spectral confocal microscope and images of TGT44 on an Olympus BX60 microscope. To determine vessel density the ratio of the CD31-stained area to the total area and the number of vessels in each area were quantified. Quantifications were carried out in six hotspot fields of viable tissue zones at 400x magnification for each tumor, using Image J software. An average value for each tumor was obtained for each variable. Results are expressed as the means for each treatment group.

### Histological study

Representative fragments of the primary and xenografted tumors were fixed in buffered formalin, dehydrated and embedded in paraffin. Tissue sections (3–4 μm) were stained with hematoxylin-eosin for morphological analysis.

Anti-EMA mouse monoclonal antibody (1:200, Dako, clone E29, M0613), anti-Cam5.2 mouse monoclonal antibody (ready-to-use, Becton Dickinson, ref 345779), anti-AFP rabbit polyclonal antibody (ready-to-use, Dako, IR500) and anti-c-KIT (CD117) rabbit polyclonal antibody (1:750, Dako, A4502) were used for immunohistochemical characterization.

Antigen retrieval was performed in the Dako PT Link using the high pH Dako retrieval solution (K8004) for AFP and c-KIT, and the low pH Dako retrieval solution (K8005) for Cam5.2 and EMA for 20 min at 95°C. The slides were stained on an Autostainer Link 48 (Dako). The EnVision^TM^Flex+ detection system (Dako, Glostrup, Denmark, K8002) was used for visualization. Sections were incubated for 5 min with peroxidase-blocking reagent (SM801), 20 min with the primary antibody, 20 min with the EnVision™ FLEX/HRP Detection Reagent (SM802), 10 min with EnVision™ FLEX DAB+ Chromogen (DM827)/EnVision™ FLEX Substrate Buffer (SM803) mix and 5 min with EnVision™ FLEX Hematoxylin (K8008). The slides were then dehydrated and mounted.

### Western blotting

Samples from two fragments of TGT44 tumor were mechanically disrupted using RIPA lysis buffer (0.1% SDS, 1% NP-40, 0.5% sodium deoxycholate, 50 mM NaF, 5 mM EDTA, 40 mM β-glycerophosphate, 200 μM sodium orthovanadate, 100 μM phenylmethylsulfonyl fluoride, 1 μM pepstatin A, 1 μg/ml leupeptin, 4 μg/ml aprotinin in PBS, pH 7.4) and a glass homogenizer on ice. Insoluble material was removed by centrifugation at 12,000 X g for 10 min at 4°C. Protein concentration was determined using a BCA assay kit (Pierce). Proteins from tumor lysates were separated on a 7.5% acrylamide-SDS gel and electrophoretically transferred to an Immobilon-P membrane (Millipore, Billerica, MA) in 25 mM Tris–HCl, 0.19 M glycine, 10% methanol. The membrane was blocked in TBS (150 mM NaCl, 50 mM Tris, pH 7.4) containing 5% non-fat dry milk for 1 h. Blots were incubated with 1/500 polyclonal goat anti-human PDGFRα antibody (RαD systems), 1/500 polyclonal rabbit anti-human PDGFRβ (P-20) antibody (Santa Cruz Biotechnology, Inc.) or 1/1000 monoclonal mouse anti-tubulin antibody (Sigma Chemical, St Louis, MO) in TBS 1% non-fat dry milk overnight at 4°C. After washing in TBS 0.1% Triton X-100, blots were incubated with 1/2500 anti-goat IgG (Dako) antibody, 1/2500 anti-rabbit IG or 1/5000 anti-mouse IG (Amersham Pharmacia Biotech, Cambridge, UK) horseradish peroxidase linked antibodies, in TBS 1% non-fat dry milk at room temperature for 1 h and after washing in TBS 0.1% Triton X-100, blots were developed with an enhanced chemiluminescence system (Amersham Pharmacia Biotech).

### Quantitative real-time PCR

Total RNA from tumors was extracted using the RNAeasy Plus Mini Kit (Qiagen) and cDNA obtained after a reverse transcription reaction (High Capacity cDNA Reverse Transcription Kit, Applied Biosystems). Real-time PCR of cDNA obtained from TGT44, TGT1, TGT38 independent tumors and the GCT27 and 1411H cell lines was done as described elsewhere [26]. Human-specific primers used were PDGFRα (5′- AGTTCCTTCATCCATTCTGGACT and 5′- CCGTCTGTCCCCCAGTT), PDGFRβ (5′-CATCACCGTGGTTGAGAGC and 5′-AATTGTAGTGTGCCCACCTCTC) and the housekeeping gene β-actin (5′GAGGCAGCCAGGGCTTA and 5′AACTAAGGTGTGCACTTTTATTCAACT).

### Statistical analysis

Statistical analysis was carried out with SPSS for Windows (version 13.0, SPSS, Inc., Chicago, IL). Statistical significance of differences in tumor growth or CD31 expression between different treatment groups was determined using the two-tail Mann–Whitney U test. In all experiments, differences were considered statistically significant for values of p < 0.05.

## Results

### TGT44 CDDP-refractory tumor model characterization

As already mentioned, the main objective of our work was to find new therapeutic possibilities not only for patients who had become resistant after CDDP treatment, but also for patients directly refractory to this treatment. In a previous article [26], we presented data obtained from a model of CDDP-resistant testicular GCT (TGT38R) generated in our laboratory after the administration of several doses of *in vivo* cisplatin. In order to generate an equivalent testicular GCT mouse model, in this case for CDDP-refractory tumors, we orthotopically implanted a human retroperitoneal metastatic mixed GCT (with teratoma and yolk sac components) that was refractory to first-line CDDP chemotherapy. The yolk sac component grew in the mice and generated TGT44. After orthotopic implantation of this primary tumor in mice, animals were subjected to CDDP treatment as a first test of CDDP resistance. No difference in time of tumor growth was observed after CDDP treatment, confirming that TGT44 retains refractivity to CDDP treatment (data not shown).

**Figure 1:**
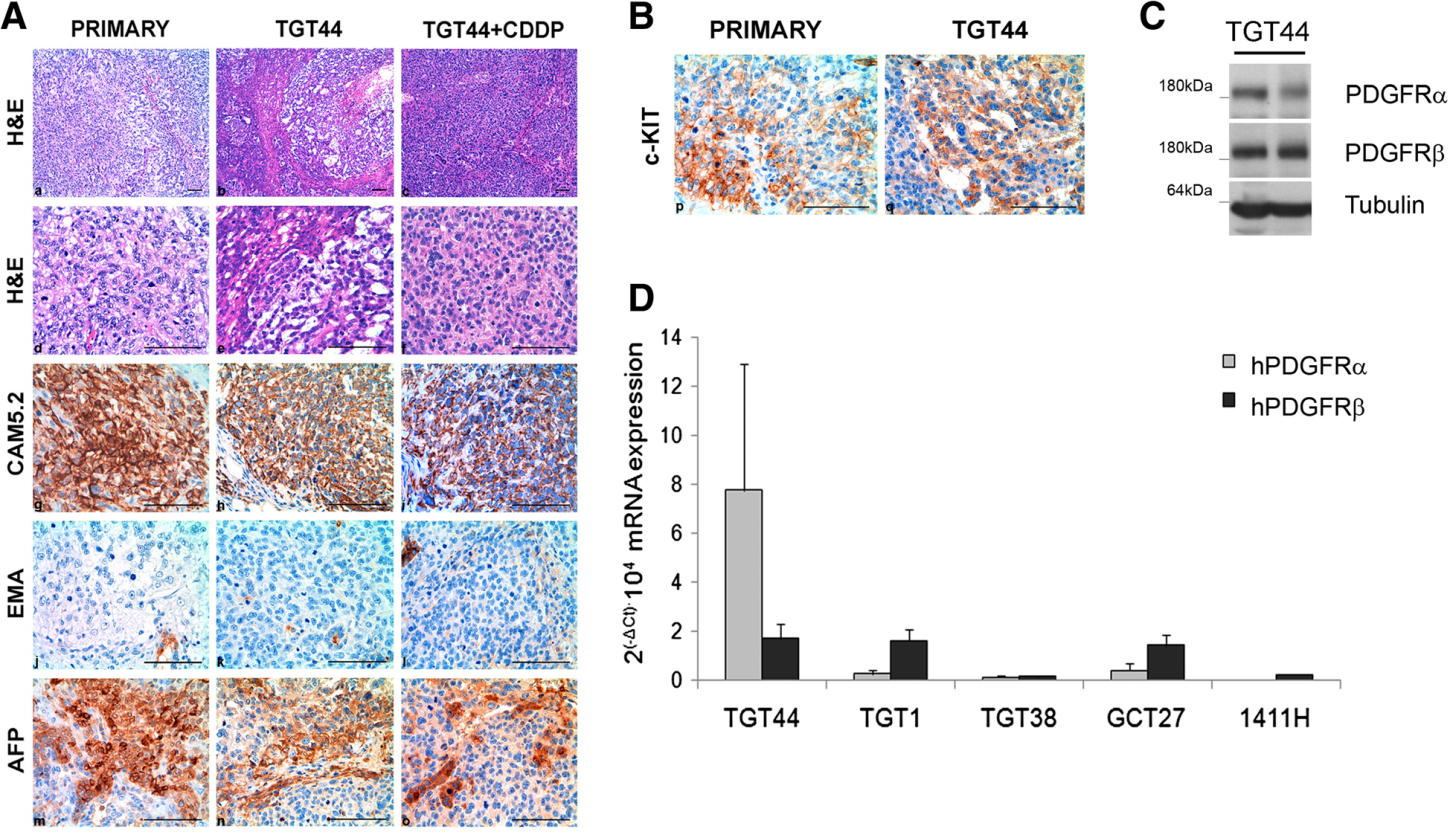
**Immunohistochemical analysis and expression of pazopanib targets in TGT44. A)** Histological and immunohistochemical characterization of TGT44, CDDP-treated TGT44 (with three injections of 4 mg/kg CDDP) and the original tumor biopsy: (a-f) Hematoxylin-eosin staining (a-c: 150x, bar 100 μm; d-f: 400x, bar 100 μm); (g-i) CAM5.2 immunostaining; (j-l) EMA immunostaining; (m-o) AFP immunostaining. 400x, bar 100 μm. **B)** c-KIT expression in TGT44 and original tumor biopsy was characterized by immunohistochemical analysis. 400x, bar 100 μm. **C)** Expression of PDGFRα and PDGFRβ receptors, and tubulin as a loading control, was analyzed by western blot of two TGT44 independent tumors. **D)** mRNA levels of human PDGFRα and PDGFRβ were analyzed by quantitative real-time PCR in TGT44, TGT1, TGT38 orthotopic tumor models, and in GCT27 and 1411H testicular cell lines. Results are expressed as 2^(−ΔCt)^·10^4^ (with SD).

A histological analysis was performed to characterize the retroperitoneal surgical specimen and to compare it with the orthotopic tumor before and after treatment with CDDP. The yolk sac component of the surgical sample, as well as of the orthotopic tumor before CDDP treatment in mice showed solid and focally microcystic patterns (Figure 1A, a, b, d and e), whereas the orthotopic CDDP-treated tumor had a predominantly solid yolk sac pattern (Figure 1A, c and f). The immunohistochemical profile was similar in the original metastasis and the two orthotopic tumors, and was characteristic of a yolk sac tumor with extensive expression of cytokeratine Cam5.2 (Figure 1A, g, h and i), but with only focal expression of EMA (Figure 1A, j, k and l) and patchy immunoreactivity for AFP (Figure 1A, m, n and o).

Our next objective was to evaluate the efficacy of pazopanib in the TGT44 CDDP-refractory model of testicular GCT. Thus, we first studied the presence of different pazopanib targets in these tumors. TGT44 presented vascular structures, positive for CD31 (an endothelial marker), but fewer of them than in, for example, choriocarcinoma tumors (Figure 1A). c-KIT tyrosine kinase receptor was detected by immunohistochemistry in the TGT44 and primary tumors (Figure 1B). Moreover, PDGFRα and PDGFRβ expression was detected by western blot in TGT44 tumors (Figure 1C), confirming that these two pazopanib targets were also present in the tumor. In order to confirm tumoral expression of these receptors, specific human PDGFRα and PDGFRβ mRNA levels were analyzed in TGT44. We also measured their levels in other orthotopic testicular tumor models, such as TGT1 and TGT38, wherein the expression of mRNAs has already described [26], and in two testicular tumoral cell lines, the embryonal carcinoma GCT27 cell line [29] and the yolk sac 1411H cell line [30]. When we compared the mRNA levels of these samples we observed that TGT44 expressed both hPDGFRα and hPDGFRβ (Figure 1D).

### Pazopanib has anti-tumor and anti-angiogenic activity in TGT44 orthotopic CDDP-refractory human tumor model

**Figure 2:**
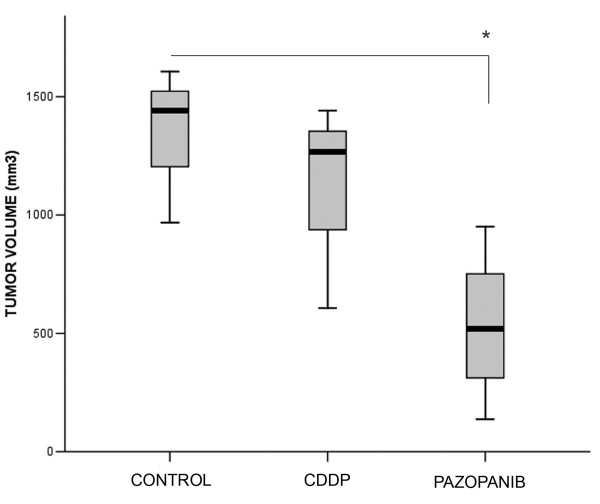
**Pazopanib inhibits tumor growth in a xenograft orthotopic model of CDDP-primary refractory yolk sac testicular germ cell tumor.** Mice with an orthotopically implanted TGT44 CDDP-refractory yolk sac tumor were treated with vehicle, four injections of 4 mg/kg CDDP or daily 100 mg/kg pazopanib for six weeks. Mice were sacrificed when control mouse tumors affected the wellbeing of the animals. Final volumes are illustrated by a boxplot. *, p < 0.05 (two-tail Mann–Whitney U test).

Having confirmed that the pazopanib targets were expressed in TGT44, mice bearing this tumor were randomized into three groups and treated with vehicle, CDDP or pazopanib. CDDP resistance was confirmed when no significant inhibition of tumor volume was observed after mice were treated with CDDP (Figure 2). However, the final tumor volume of the mice treated with pazopanib was significantly smaller than in the control group (Figure 2).

**Figure 3:**
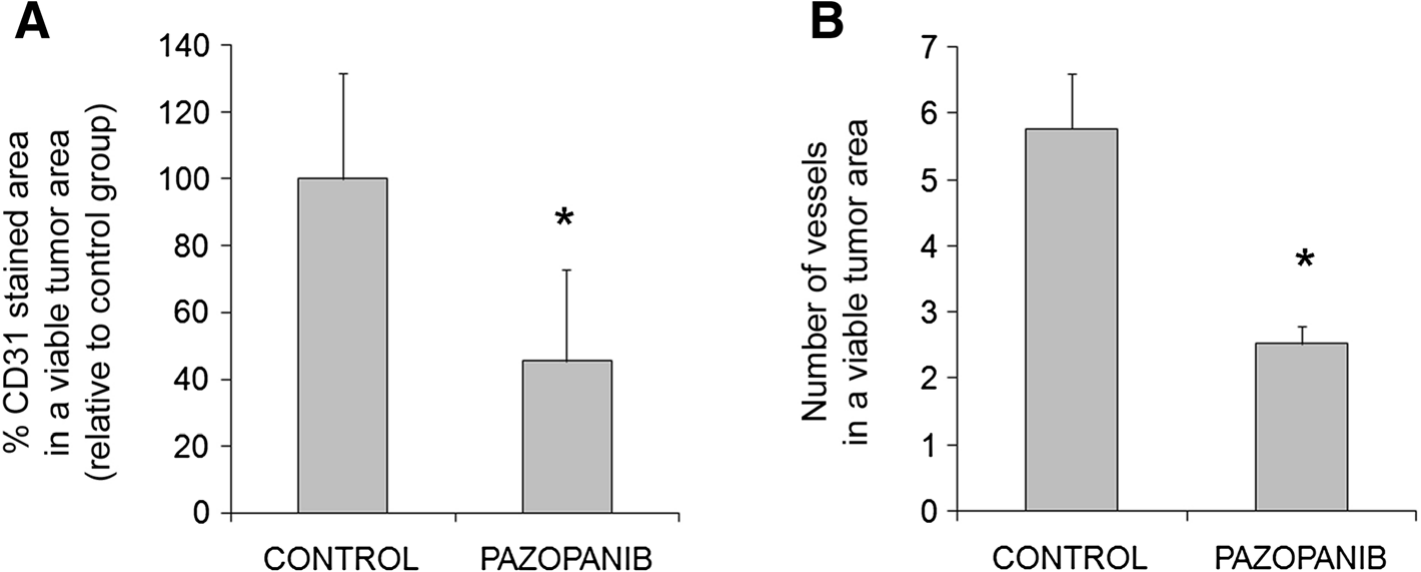
**Pazopanib displays antiangiogenic activity in a CDDP-refractory model of testicular GCT. A)** Percentage CD31-stained area in a viable tumor area and **B)** number of vessels in a viable tumor area were quantified using Volocity software. Means and SDs of six sections of each tumor were determined as the positive CD31-stained area relative to the control group. *, p < 0.05 (two-tail Mann–Whitney U test).

Sections of tumors were further subjected to CD31 staining to evaluate the tumor vascular endothelium. The ratio of the CD31-stained area to the total area of tumor sections from both treatment groups (Figure 3A) were analyzed, as well as the number of vessels in a viable tumor area (Figure 3B). Pazopanib induced a significant reduction in tumor vascular density and the number of vessels in TGT44, confirming its anti-angiogenic activity in the TGT44 tumor model.

### Pazopanib inhibits tumor growth and synergizes with lapatinib anti-ErbB treatment in an orthotopic model of testicular choriocarcinoma

**Figure 4:**
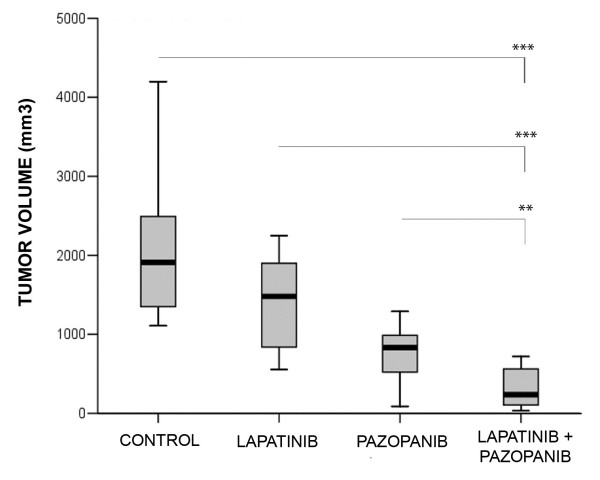
**Pazopanib inhibits tumor growth in a xenograft orthotopic model of choriocarcinoma testicular germ cell tumor and synergizes with lapatinib.** Mice orthotopically implanted with the TGT38 choriocarinoma were treated daily with vehicle, 100 mg/kg pazopanib, 100 mg/kg lapatinib or a combination of both for 14 days. Final tumor volumes are illustrated by a boxplot. **, p < 0.01; ***, p < 0.001 (two-tail Mann–Whitney U test). All treatments showed significant inhibition with respect to the control group.

We recently showed that testicular cancer cells are very sensitive to dual anti-ErbB1 and anti-ErbB2 inhibitors such as lapatinib, in contrast with the very weak effect obtained with pure anti-ErbB1 inhibitors [31]. We found the same effect *in vivo* in an orthotopic model of human choriocarinoma [31]. To establish whether there was any synergistic effect of pazopanib and lapatinib, we selected the same model, TGT38, described by Castillo-Avila et al. [26], which reproduces the histological and genetic characteristics of the original choriocarcinoma primary testicular tumor. Mice with orthotopically implanted TGT38 were treated with vehicle, pazopanib, lapatinib or the pazopanib/lapatinib combination. These treatments had no significant effect on mouse body weight or toxicity in liver and the animals appeared healthy and active throughout the study (Additional file 1: Figure S1). Their tumor volumes were determined at the end of the experiment (Figure 4). As previously described, lapatinib treatment caused a significant decrease in tumor volume relative to the control group. Pazopanib treatment also significantly inhibited the increase in tumor volume compared with the control group. The effect of treating the animals with both inhibitors was greater than when the inhibitors were administered separately (Figure 4). Moreover, values of the combination ratio (CR) were greater than 1, indicating that the combination treatment had supra-additive effects.

### Pazopanib reduces tumor vascular density

**Figure 5:**
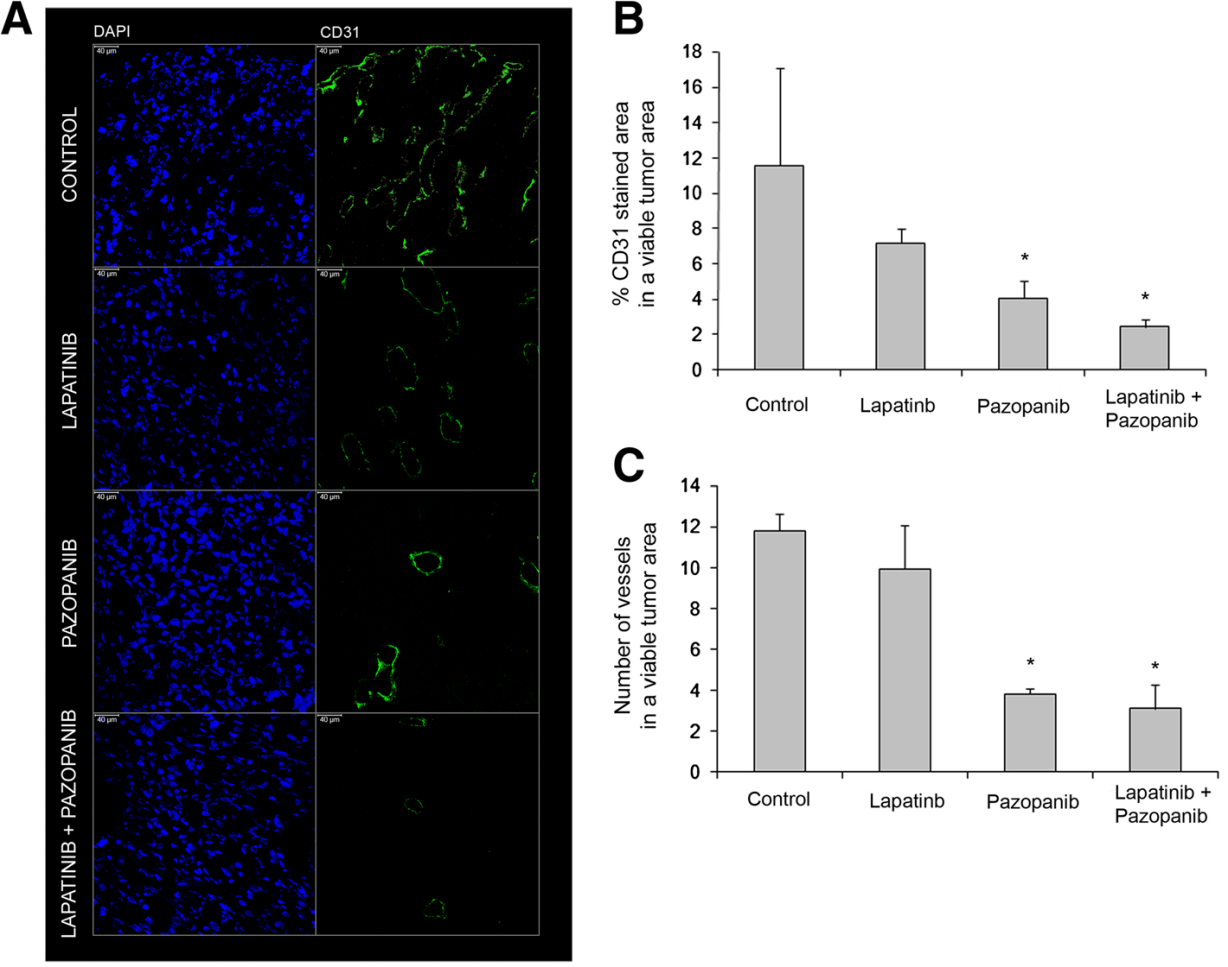
**Pazopanib reduces vessel density in a xenograft orthotopic model of choriocarcinoma testicular germ cell tumor. (A)** Staining for CD31 endothelial marker was carried out in viable zones of TGT38 choriocarcinoma tumors implanted in mice and treated as in Figure 4. Bar 40 μm, 400x. CD31 expression was analyzed by measuring the percentage of area with positive CD31 staining in a viable tumor area **(B)** and the number of vessel structures **(C)**. Quantification was carried out using Image J software. The means and SDs of six sections of each tumor (four tumors per treatment group) of each variable are shown. *, p < 0.05 (two-tail Mann–Whitney U test).

To assess the effects of the different inhibitors on tumoral vasculature, the tumoral vascular endothelium was evaluated by immunofluorescence staining for the endothelial marker CD31 (Figure 5A). The percentage of CD31-stained area to the total tumor area (Figure 5B) and the number of vessels in viable tumor zones (Figure 5C) were measured. Lapatinib treatment did not significantly affect either of these characteristics. In contrast, pazopanib treatment caused a significant decrease in both variables, the effect being maintained when pazopanib was administrated with lapatinib.

## Discussion

Our results show that pazopanib as a single agent has anti-tumor and anti-angiogenic activity in preclinical models of CDDP-sensitive and CDDP-refractory testicular GCTs. Its combination with the dual anti-ErbB1 and anti-ErbB2 inhibitor lapatinib had a synergistic effect on tumoral growth. These results further confirm and extend our previous results with sunitinib [26]. Nevertheless, it is important to stress that the previous study showed sunitinib efficacy in a CDDP-resistant xenograft GCT model (TGT38R). That model was generated in our laboratory by prolonged CDDP treatment of mice bearing the primary tumor. In contrast, the CDDP-resistant testicular tumor model used in this study (TGT44) came from a patient with a CDDP-refractory metastatic testicular tumor. We have shown that this tumor retained CDDP resistance after transfer from the patient to the orthotopic animal model. Moreover, no significant histological differences were observed between the primary and the orthotopically implanted tumor, even after treatment with CDDP. Thus, this new testicular *in vivo* tumor model offers new possibilities for comparing as yet undiscovered mechanisms involved in *de novo* resistance in patients with acquired resistance.

Pazopanib kinase selectivity (IC_50_) shows a specific pattern, with similarities to other TKIs such as sunitinib (inhibition of c-KIT and PDGFRα and β) sorafenib (inhibition of c-Raf) or both (inhibition of VEGFRs) [32]. Currently, pazopanib is used as a second-line treatment in patients with clear-cell RCC that relapses after the administration of sunitinib or bevacizumab [24]. The efficacy of pazopanib compared with that of sunitinib remains unclear, although there is an ongoing clinical trial comparing the effects of the two drugs in locally advanced and/or metastatic RCC in patients with no prior treatment [24]. However, given the specific kinase inhibition pattern of pazopanib compared with that of sunitinib or sorafenib, it would be interesting to assay the effects of this drug in different tumors at the preclinical and clinical stages. The present study shows that pazopanib as a single agent is also effective and significantly inhibits growth of two different testicular GCTs orthotopically grown in nude mice, a cisplatin-sensitive choriocarcinoma and a yolk sac metastatic cisplatin-refractory tumor. This growth inhibition is associated in both tumors with a reduction in tumor vessel density, clearly indicating an anti-angiogenic effect. Moreover, in our xenografts, tumoral testicular cells also express some of the pazopanib targets, such as c-KIT and PDGFR α and β in TGT44, and both PDGFRs in TGT38, which also suggests a direct anti-tumoral effect in our *in vivo* models. In fact, cell cultures of testicular cancer cells sensitive or resistant to cisplatin respond to pazopanib by blocking cell growth (data not shown), confirming this direct anti-tumoral effect. Taken together, our results indicate that pazopanib probably influences tumoral growth by a combination of effects comprising indirect anti-angiogenic and direct anti-tumoral activity in testicular cells.

The treatment of relapsed or CDDP-refractory GCT patients remains a clinical challenge. The options for these patients include surgery, radiotherapy and the use of conventional-dose or high-dose chemotherapy, but their prognosis is generally poor [4,5], highlighting the need for new, alternative therapies. Anti-angiogenic therapy has been proposed as a strategy for treating testicular GCTs [11,33], and successful results have already been obtained in preclinical models treated with sunitinib, as reported by Castillo-Ávila et al. [26] and Oechsle et al. [34], or with other anti-angiogenic compounds [35,36]. Sunitinib as a single agent was tested in two clinical trials of refractory GCT [34,37], giving modest results, with only a few cases of short-duration disease stabilization followed by rapid progressive disease in one study [37], but with three temporary partial responses (9%) and 41% of cases of stable disease [34] in the other. Moreover, there was a decrease in the frequency of tumor markers following sunitinib treatment, suggesting that the targets of sunitinib may still be important to GCT biology [37]. In fact, a recent study assessing the efficacy of the combination of oxaliplatin and bevazucimab recorded a substantial number of responses, clearly more than found in previous studies in which oxaliplatin alone was used [38]. This suggests that the use of compounds targeting VEGF might enhance the performance of chemotherapy in the treatment of GCTs. It is important to point out that both tumors analyzed in the present study, in which pazopanib is shown to be effective, were both positive for some pazopanib targets. This suggests that only those patients whose tumors are positive for the specific targets of these inhibitors may benefit from their effects.

Our results also show a clear synergistic effect of pazopanib when administered in combination with lapatinib, a dual anti-ErbB1 and anti-ErbB2 inhibitor. As we previously described [31], lapatinib alone partially blocks tumor growth, but does not affect angiogenesis. In contrast, pazopanib alone or in combination with lapatinib has the same anti-angiogenic effect, ruling out the possibility of an indirect anti-angiogenic effect arising from anti-ErbB therapy in this model. This result, and the observed synergistic effect on tumor volume, indicates independent targets and effects on tumoral growth for both inhibitors. A similar effect has been seen when inhibitors for all pathways were combined, for example in xenograft models of head and neck tumors [39], non-small cell lung cancers [40] and in breast cancer brain metastases [41]. Some dual anti-VEGFR and anti-ErbB inhibitors, such as vandetanib [42], AEE788 [43] and SKLB1206 [44], have been developed and assayed, with promising results. The combination of lapatinib and pazopanib has also been assayed in various carcinoma cell lines and shown to have synergistic proapoptotic effects [45]. In contrast, phase II clinical trials in cervical cancer and ErbB2-positive breast cancer patients detected toxicity when the two drugs were combined [46,47]. In the near future it will be essential to determine whether less toxic combinatory doses are also effective in patients.

## Conclusions

Although the true activity of the various VEGFR inhibitors in GCTs remains to be demonstrated [37], we believe that pazopanib is potentially a new agent that merits clinical testing in CDDP-refractory GCT patients as a single agent or in combination with other therapies, such as ErbB-targeted therapies.

## Competing interests

The authors declare that they have no competing interests.

## Authors’ contributions

MJ carried out the experimental part of this study and helped write the manuscript. A Vidal carried out the immunohistochemistry and was involved in drafting the manuscript. XGM helped design the study. JMP helped design the study. EC helped design the study. OC helped design the study and drafted the manuscript. MG helped design the study and drafted the manuscript. JRG helped design the study. A Villanueva generated the mouse models used in this study and helped write the manuscript. FV helped design the study, draft and write the manuscript. All authors have read and approved the final manuscript.

## Pre-publication history

The pre-publication history for this paper can be accessed here:

http://www.biomedcentral.com/1471-2407/13/382/prepub

## Supplementary Material

Additional file 1: Figure S1 A) Mouse body weight throughout treatment. B) Apoptotic cell detection by TUNEL staining was performed in liver sections from control and combined drug treatment. Positive TUNEL cells are pointed with black arrows. Results obtained showed no difference between control and treated conditions and all liver samples presented ratios of 0.1-0.5 ‰ positive TUNEL cells. TGT38 tumor treated with lapatinib was used as positive control. Bar 50 μm, 400X. Click here for file
