# Supplementary figures and images for: Effectivity of pazopanib treatment in orthotopic models of human testicular germ cell tumors
Source: BMC Cancer. 2013 Aug 10;13:382. doi: 10.1186/1471-2407-13-382 (PMC3751347; doi:10.1186/1471-2407-13-382)

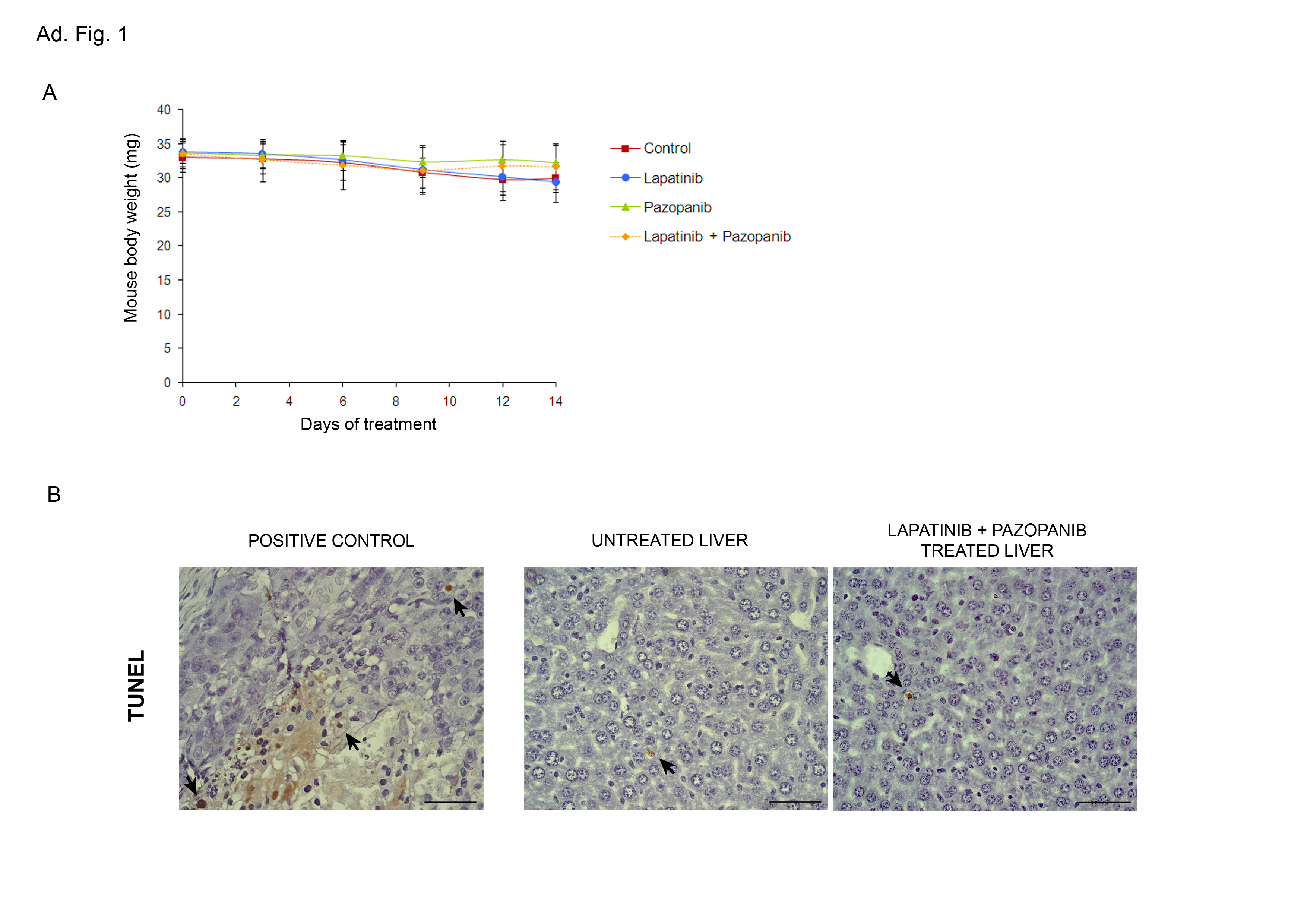

Supplement: Additional file 1: Figure S1 — A) Mouse body weight throughout treatment. B) Apoptotic cell detection by TUNEL staining was performed in liver sections from control and combined drug treatment. Positive TUNEL cells are pointed with black arrows. Results obtained showed no difference between control and treated conditions and all liver samples presented ratios of 0.1-0.5 ‰ positive TUNEL cells. TGT38 tumor treated with lapatinib was used as positive control. Bar 50 μm, 400X. [file 1471-2407-13-382-S1.jpeg]
